# The Immunological Mechanisms and Immune-Based Biomarkers of Drug-Induced Liver Injury

**DOI:** 10.3389/fphar.2021.723940

**Published:** 2021-10-15

**Authors:** Wenhui Liu, Xiangchang Zeng, Yating Liu, Jinfeng Liu, Chaopeng Li, Lulu Chen, Hongying Chen, Dongsheng Ouyang

**Affiliations:** ^1^ Department of Clinical Pharmacology, Xiangya Hospital, Central South University, Changsha, China; ^2^ Hunan Key Laboratory of Pharmacogenetics, Institute of Clinical Pharmacology, Central South University, Changsha, China; ^3^ Engineering Research Center of Applied Technology of Pharmacogenomics, Ministry of Education, Changsha, China; ^4^ National Clinical Research Center for Geriatric Disorders, Changsha, China; ^5^ Hunan Key Laboratory for Bioanalysis of Complex Matrix Samples, Changsha Duxact Biotech Co., Ltd., Changsha, China

**Keywords:** drug-induced liver injury, mechanism, immune response, immune, biomarker

## Abstract

Drug-induced liver injury (DILI) has become one of the major challenges of drug safety all over the word. So far, about 1,100 commonly used drugs including the medications used regularly, herbal and/or dietary supplements, have been reported to induce liver injury. Moreover, DILI is the main cause of the interruption of new drugs development and drugs withdrawn from the pharmaceutical market. Acute DILI may evolve into chronic DILI or even worse, commonly lead to life-threatening acute liver failure in Western countries. It is generally considered to have a close relationship to genetic factors, environmental risk factors, and host immunity, through the drug itself or its metabolites, leading to a series of cellular events, such as haptenization and immune response activation. Despite many researches on DILI, the specific biomarkers about it are not applicable to clinical diagnosis, which still relies on the exclusion of other causes of liver disease in clinical practice as before. Additionally, circumstantial evidence has suggested that DILI is mediated by the immune system. Here, we review the underlying mechanisms of the immune response to DILI and provide guidance for the future development of biomarkers for the early detection, prediction, and diagnosis of DILI.

## Introduction

Drug-induced liver injury (DILI) is usually caused by regular medications, herbal and dietary supplements (HDS), manifested as liver damage caused by the drug itself or its metabolites. It has become the main reason for the interruption of drug development during clinical research as well as drugs withdrawal from the pharmaceutical market (Wysowski and Swartz, 2005). The incidence of DILI is 2.7–14 per 100,000 cases in Europe and the United States (Lei et al., 2015; Wang J. et al., 2015; Navarro et al., 2017), while that is about 23.8 per 100,000 cases in mainland China (Shen et al., 2019). In the United States, DILI is account for more than 50% of patients with acute liver failure (ALF) (European Association for the Study of the Liver, 2019). Acute DILI could evolve into chronic liver injury and reach hepatic failure, which requires liver transplantation, or even lead to death (Hayashi and Bjornsson, 2018). Therefore, DILI occurs throughout the life cycle of drug development and post-marketing scenarios, and it has become one of the major life-threatening public health events.

DILI is divided into intrinsic and idiosyncratic DILI (IDILI) according to the pathogenesis (Fontana, 2014). Intrinsic DILI, a predictable and rapid onset of liver injury in a dose-dependent manner, could be reproduced in animal models (Chalasani et al., 2014). In contrast, IDILI, an unexpected type of liver injury is mainly affected by a host of factors and accounts for 11% of ALF in the United States (Lucena et al., 2020). DILI is affected by multiple factors, including environmental exposures (drug dose, lipid solubility, drug interactions, and others) and genetic factors (drug metabolic enzymes, transporters, nuclear receptors, human leukocyte antigen (HLA), and others) (Björnsson, 2014; Chalasani and Björnsson, 2010). Age, gender, and pregnancy also influence the progression of liver injury caused by certain drugs (Dugan et al., 2011). At present, the clinical diagnosis of DILI is still based on biochemical detection of alanine aminotransferase (ALT) and aspartate transaminase (AST), and liver biopsy, still as the gold standard for confirmatory diagnosis. However, these methods not only lack specificity but also easily cause the host damage. Moreover, these cannot determine which drug should be responsible for liver injury given a the scenario of a combination of multiple drugs. Therefore, the development of mechanism-based specific biomarkers is significant for the prediction and diagnosis of DILI.

**FIGURE 1:**
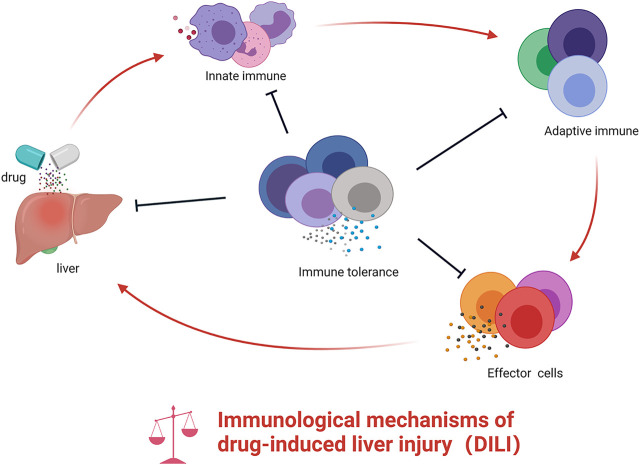
The role of innate immunity and adaptive immunity in DILI. Drug or their reactive metabolites lead to cell stress, damage, or death, which release some molecules that recruit and activate innate immune cells prompting the release of pro-inflammatory cytokines. These mediators stimulate adaptive immune cells, ultimately resulting in the activation of T cells into effector cells and B cells into plasma cell–released antibodies. During the activation of innate and adaptive immunity, the host immune tolerance–related immune cells or cytokine may exert immunosuppressive effects. However, if the balance is broken, this will further aggravate the inflammatory response in the liver.

Existing studies have shown that mitochondrial dysfunction, oxidative stress, the imbalanced production and degradation of bile acid, and inflammatory responses are involved in the occurrence and development of DILI (Li et al., 2017; Kakisaka et al., 2018; Tu et al., 2019; Ejigu and Abay, 2020). However, these findings cannot fully elucidate the mechanism of DILI. The liver as an immune organ gathers several subsets of innate immune cells (e.g., neutrophils, macrophages, dendritic cells (DCs), natural killer (NK) cells, lymphoid cells, γδT cells, and others) and adaptive immune cells (such as T cells and B cells) (Ostapowicz et al., 2002; Adams et al., 2010) (Figure 1). Furthermore, the abnormalities of the above-mentioned immune cells and associated molecules affect the status of acute liver injury (Vega et al., 2017; García-Cortés et al., 2018; Stravitz and Lee, 2019). Recently, numerous clinical and experimental studies have found that immune responses are closely related to the development of DILI (Woolbright and Jaeschke, 2018). In particular, HLA alleles have been reported strongly associated with liver injury caused by a series of other drugs, e.g., flucloxacillin, clavulanic acid-amoxicillin, and *Polygonum multiflorum*, providing new insights for uncovering the mystery of DILI. In addition, some HDS-induced liver injuries have commonly observed antibodies or active T cells that support the immune system play a key role in the pathogenesis of liver injury (Manso et al., 2011; Wang et al., 2019). Here, we integrate the progress of the immune mechanisms of DILI and provide a reference for the prevention and treatment of DILI.

## The Innate Immune System and Drug-Induced Liver Injury

The innate immune system is the first line of host defense, and its activation is much more rapid than the adaptive immune system (Patel et al., 2016). The innate immune system is a natural immune defense system, which is gradually formed in the long-term phylogenetic evolution of organisms, and is mainly composed of tissue barriers, innate immune cells, and molecules. Drugs or active metabolites could form new antigens to initiate innate immune responses and ultimately mediate immune liver damage. Damaged hepatocytes release related danger-associated molecular patterns (DAMPs) to activate innate immune cells, which secrete related cytokines and chemokines that recruit amounts of neutrophils and monocytes to the injured sites to clear necrotic cell debris. DAMPs include high-mobility group box protein 1 (HMGB1), S100 proteins, heat shock proteins, ATPs, and some others like mitochondria-derived DAMPs, reported to induce inflammasomes via binding Toll-like receptors 2 and 4 (Vénéreau et al., 2015). In turn, under certain conditions, the recruited immune cells could further aggravate liver injury by releasing a large number of pro-inflammatory factors to form a cytokine storm. Many other immune cells and the cytokines related to the innate immune response are involved in the occurrence of DILI, such as Kupffer cells (KCs), macrophages, type-1 innate lymphoid cells (ILC1s), NK cells, neutrophils, etc. These cells promote the inflammatory response by producing cytokines, chemokines, and reactive oxygen species (ROS) (Friedman et al., 2018), which in turn recruit immune cells to the site of injury to control the damage and initiate the adaptive immune response.

### Kupffer Cells

KCs, as a specific type of macrophages residing in the liver, play a key role in immune-mediated liver injury (Dixon et al., 2013; Akai et al., 2016; Rose et al., 2016). Generally, KCs are divided into two types, M1 and M2. M1 KCs secrete pro-inflammatory cytokines, e.g., IL-1β, IL-6, tumor necrosis factor alpha (TNF-α), which determine the inflammatory signaling pathway (Liu et al., 2015; Seo et al., 2018). However, M2 KCs have a weak antigen presentation ability and secrete potent immunosuppressive factors including IL-10 (Zeng et al., 2016). As an important subset of innate immunity in the liver, KCs could recognize danger signal molecules to scavenge dead liver cells (Li et al., 2017). During liver injury, KCs are complemented by infiltrating macrophages expressing distinct surface markers (Holt et al., 2008), up-regulating mitogen-activated protein kinase, and increasing the release of pro-inflammatory cytokines, which are important intermediate steps for immune-mediated liver injury (Zhang et al., 2019). Furthermore, KCs are also considered to exacerbate liver injury by oxygen free radicals (Michael et al., 1999). Interestingly, in KC-depleted mice, the increased hepatotoxicity under acetaminophen (APAP) exposure suggested that KCs appear to have an effect of hepatoprotection (Ju et al., 2002), maybe due to the decreased expression of several hepatoregulatory cytokines like IL-10, an anti-inflammatory cytokine.

### Dendritic Cells

In the liver, DCs as the main antigen-presenting cells (APCs) regularly divide into two subsets, classical DCs (cDCs) and plasmacytoid DCs (pDCs), and could initiate both innate and adaptive immune responses (Plitas et al., 2008; Steinman, 2008). cDCs as APCs express high levels of major histocompatibility complex (MHC)-II, while pDCs have a limited ability to present antigens due to their expressing relatively lower levels of MHC-II (Rahman and Aloman, 2013). Studies have shown that immaturity in liver DCs commonly mediates tolerance rather than immunogenicity as in the steady-state liver (Xia et al., 2008). DCs as hepatic immune system sentinels are a significant subset of the non-hepatocytes to alert the immune system during the presence of harmful pathogens. DCs also produce large quantities of cytokines, such as interferon-gamma (IFN-γ), promoting the activation of cytotoxic T cells. The mechanism of DILI is thought to be related to the interaction between APCs and T cells because of the damaged hepatocytes leading to the release of DAMPs, toxic components, and reactive metabolites, which aggravates liver injury and eventually results in liver failure (Grove and Aithal, 2015). Connolly et al. (2011) found that the immune-phenotype of DC in the liver significantly altered after APAP exposure, which expressed higher MHC-II and co-stimulatory molecules and increased the release of monocyte chemotactic protein-1, IL-6, and TNF-α. However, studies show that DCs may play a suppressed role in inflammatory response and reduce liver injury by releasing IL-10 (Krug et al., 2001). The trigger in the hepatic microenvironment that stimulates DC in diverse states of liver injury is undefined, but it may be the key component to understanding liver immunity.

### Neutrophils

Neutrophils, as the most abundant type of innate immune cells, originate from the bone marrow and play a multifaceted role in host defense by phagocytosis, ROS, degranulation, and neutrophil extracellular trap for inflammatory responses (Jaeschke and Hasegawa, 2006; Döring et al., 2017). However, abnormal accumulation of neutrophils can lead to unexpected injury to host organs including DILI. Recently, more studies have defined that neutrophil activation commonly causes DILI, in which mitochondrial DNA originates from injured hepatocytes that could further activate neutrophils (You et al., 2006; Moles et al., 2014; Williams et al., 2014; Yuan et al., 2019). Liver injury increased expressions of chemokines, cytokines, and other immune molecules that could regulate neutrophil recruitment and activation, which cause cytotoxicity and hepatocyte death (Ramaiah and Jaeschke, 2007). In acute liver injury, activated neutrophils migrated into the hepatic parenchyma and recruited to the hepatic sinusoids can promote oxidative stress and necrosis, and further cause liver failure (Saiman and Friedman, 2012). It has been demonstrated that (Jaeschke, 2000; Fu et al., 2011) triptolide (TP) can cause stress response, lipid peroxidation, and hepatocyte necrosis, all of which can trigger neutrophil infiltration to further aggravate liver injury. Liu and Kaplowitz (2006) found that depletion of neutrophils could decrease serum ALT levels and centrilobular necrosis, in addition, ameliorate the progression and severity of APAP-induced liver injury. However, neutrophils caused host inflammatory response that could facilitate liver recovery by removing cell debris. Thus, the role of neutrophils remains controversial in DILI (Lawson et al., 2000).

### Eosinophils

Eosinophils derive from myeloid cells, with high granulated shape and secreting cytokines and enzymes to kill pathogens or host cells (Kita, 2011). Eosinophilia has been often associated with DILI, including acetaminophen, diclofenac, carbamazepine, enalapril, and halothane (Dertinger et al., 1998; Pham et al., 2001; Aithal et al., 2004; Björnsson et al., 2007; da Silva et al., 2010). The report of severe eosinophilic hepatitis in patients treated with lamotrigine showed several features of hypersensitivity, including fever, rash, lymphadenopathy, eosinophilia, pneumonitis, increased liver enzyme levels, and the eosinophilic infiltration on biopsy examination (Fix et al., 2006). Drug reaction in liver injury with eosinophilia and systemic symptoms might be one type of DILI, with a different spectrum of culprit drugs. In a study of the mouse model (Proctor et al., 2013), eosinophils infiltrated the liver during early phase of halothane-induced liver injury by the secretion of pro-inflammatory cytokines, which increased proportionally to the hepatocellular damage (Kanda et al., 2009).

### Natural Killer Cells

In humans, NK cells constitute 30–50% of hepatic lymphocytes (Nemeth et al., 2009; Qiao et al., 2018), and play a role in inspecting transformed or infected cells via the release of granzyme and perforin (Prager et al., 2019). In the liver, NK cells relate to physiological and pathophysiological processes, such as viral infections and other injuries, and participate in innate immune responses, cell-mediated cytotoxicity, as well as exocytosis of cytotoxic granules (Fasbender et al., 2016). These hepatic innate immune cells could participate in the pathogenesis of DILI. A double-stranded RNA viral mimetic that incurred the accumulation and activation of NK cells increased the halothane-induced hepatotoxicity in the mice model (Cheng et al., 2009). Indeed, NK cells can modulate DILI by IFN-γ production, resulting in hepatocytes cytotoxicity (Fasbender et al., 2020), and the cytotoxicity of NK cells are controlled by a sophisticated regulation of activating and inhibitory receptors (Godfrey et al., 2010; Bernardini et al., 2014). Evidence exist for the participation of NK cells in DILI, often involved in DNA damage, making histiocytes susceptible to NK cell lysis (Raulet et al., 2013). Studies also showed that the activation of NK cells have been claimed to be a key component in APAP-induced hepatotoxicity (Liu et al., 2004), and IFN-γ, a major source from hepatic NK cells, which has been shown to mediate immune cells infiltration, chemokine, and cytokine release and lead to hepatocyte apoptosis (Ishida et al., 2002). IFN-γ has also influenced APAP and concanavalin A–induced liver injury in animal models (Tagawa et al., 1997). Administration of exogenous IFN-γ to patients with APAP caused elevated liver enzyme in the serum, implying that IFN-γ promotes liver injury in humans (Kenna et al., 1988). In a recent study, primary human hepatocytes exposed to 148 drugs of relevant concentrations in clinical by genome-wide analysis, found that several drugs, such as valproic acid, promethazine, ketoconazole, isoniazid, activated ligands for NK cell receptors like NKp30 ligand and NKG2D ligands and incurred hepatocyte killing by NK cells (Fasbender et al., 2020). The above studies support that NK cells activation can modulate DILI by IFN-γ production and interaction with hepatocytes.

### Natural Killer T Cells

Natural killer T (NKT) cells, a unique subset of T cells, expressing both NK cell receptors and T cell receptors, are MHC class I–like molecules, CD1d-restricted and glycolipid antigen reactive (Wallace et al., 2009), and bridge innate and acquired immunity (Van Kaer et al., 2011), and are closely related to immune liver injury (Zheng et al., 2018). Distinct from conventional T lymphocytes, NKT cells preferentially taking the liver as their home, play a pathogenic role in various types of liver disease (Martin-Murphy et al., 2013; Schrumpf et al., 2015; Bandyopadhyay et al., 2016), secrete cytokines like IFN-γ, IL-4, and IL-17, and regulate the balance of pro- and anti-inflammatory responses in liver diseases (Wang and Yin, 2015; Bhattacharjee et al., 2017; Li and Hua, 2017). One case report on analyzing the hepatic and peripheral blood lymphocytes in two patients with drug-induced fulminant hepatic failure indicated that NKT cells might be involved in hepatic injury (Miyakawa et al., 2005). Studies have found that NKT cells are dominantly releasing IFN-γ and recruiting neutrophils and macrophages, leading to TP-induced liver injury (Wang et al., 2018). However, NKT cells are not only beneficial in APAP-mediated acute liver injury but also can limit inflammatory cytokine secretion (Kwon et al., 2014) because of type I and II cytokines secretion, making them both protective and harmful.

### Type-1 Innate Lymphoid Cells

Innate lymphoid cells (ILCs) were originally found as liver-resident ILCs and characterized by the lack of receptors of B cells and T cells (Spits and Di Santo, 2011; Spits et al., 2013). ILCs sense pro-inflammatory cytokines at local tissue damage sites and immediately initiate innate immune responses in tissues (Nabekura et al., 2020). According to surface markers and secreted cytokines, ILCs are divided into three subsets: ILC1s, ILC2s, and ILC3s. In multiple tissues, ILCs can orchestrate homeostasis through multiple immune cell types (Eberl et al., 2015). Besides, activated ILC1s secrete IFN-γ and can upregulate Bcl-xL to inhibit acute liver injury (Nabekura et al., 2020). However, the immune regulation triggered by ILCs also contributes to the host protection against tissue repair, metabolism, and homeostasis (Klose and Artis, 2016; Ebbo et al., 2017; Colonna, 2018). At the same time, many studies have found that innate immune cells can repair acute liver injury under appropriate conditions (Hossain and Kubes, 2019), but the related mechanisms remain unclear.

## The Adaptive Immune System and Drug-Induced Liver Injury

The adaptive immune response consists of humoral immunity mediated by B cells producing antibodies and of cellular immunity mediated by T cells (Shuai et al., 2016). In the liver, the adaptive immune system is indispensable in the pathophysiological processes of acute injuries (Gantner et al., 1995; Ke et al., 2013; Wang et al., 2015a). Many IDILI features with delayed onset and drug reactivation suggested that adaptive immune response could attack the liver and modulate individual susceptibility to liver injury (Chen et al., 2015). Some DILI patients upon drug discontinuation may develop persistent liver damage via releasing danger signals and activating innate and adaptive immune. Kim et al. (2015) found specific T cells infiltrated in amoxicillin–clavulanate–induced liver injury that seemed to be related to lymph toxin, resulting in the activation of IL-6 in the liver (Tumanov et al., 2009). However, due to the lack of valid animal models, in-depth studies of the immune mechanisms in DILI were virtually impossible.

### T Cells

T cells differentiate and mature in the thymus and then migrate to the surrounding lymphoid tissue. According to the features and surface marks, T cells can be mainly divided into two subsets, CD4^+^T and CD8^+^T cells. But according to their function, these include cytotoxic T cells (CTLs), T helper cells (Th), regulatory/suppressor T cells (Treg), and delayed hypersensitive T cells. The liver histology of IDILI most commonly infiltrates some immune cells, often including CD8^+^T cells (Mockenhaupt, 2014; Foureau et al., 2015). In addition, neoantigens by drug or its reactive metabolites in hepatocytes are presented by APCs and activate numerous CD8^+^T cells in IDILI (Foureau et al., 2015). Simultaneously, study found that CD8^+^T cells infiltrated in the liver of patients with flucloxacillin-DILI (Wuillemin et al., 2014). CTLs could kill target cells in different ways, such as by the activation of death receptors, granule exocytosis, and release of cytokines (Pinkoski et al., 2001; Metkar et al., 2003). Fulminant liver failure is probably caused by the infiltration of CTLs in drug adverse reactions (Amante et al., 2009). The infiltration of granzyme B^+^CD3^+^T cells was found around the apoptotic hepatocytes in patients with fulminant liver failure after vancomycin intake (Mennicke et al., 2009). Several MHC alleles associated with the susceptibility to IDILI present neoantigens to stimulate the activation and proliferation of T cells in IDILI (Daly and Day, 2012). The MHC-I molecules expressed on all nucleated cells present endogenous peptides for CD8^+^T cells while the MHC-II molecules present exogenous peptides for CD4^+^T cells (Jongsma et al., 2019). In addition, many HLA predict the risk of IDILI associated with MHC-I (Daly and Day, 2012), which also suggests that CD8^+^T cell–mediated adaptive immune response causes most of IDILI. So, drug or drug metabolites modify proteins likely through the specific MHC molecule–initiated immune response.

### B Cells

B lymphocytes stem from the bone marrow, and their size is slightly larger than that of T lymphocytes. Mature B cells emigrate from the peripheral blood and enter the spleen and lymph nodes. Following activation, B cells become plasma cells and produce antibodies participating in immune response. Recently, antidrug antibodies were observed in patients with amodiaquine, which suggests that the idiosyncratic drug reactions are immune mediated (Daly and Day, 2012). Most cases of severe isonicotinic acid hydrazide (INH)–induced liver injury were associated with antibodies against INH-modified proteins and native proteins, especially the cytochrome P450 (CYP) (Metush et al., 2014). The serum samples from patients with nomifensine were screened by immunoassay for IgE and IgG antibodies, and all patients had specific IgG antibodies (Wälti et al., 1983). In addition, patients with halothane-developed IDILI were found to have some antibodies against trifluoroacetylated proteins as well as autoantibodies against native proteins (Martin et al., 1993). The existence of antibodies can only indicate that DILI is immune-mediated, but there is no exact evidence to support that antibodies are the culprit of DILI. They could be explained as an immune response to liver damage or even to resolve the immune response.

## The Immune Tolerance and Drug-Induced Liver Injury

The liver is an immune-privileged organ with complex immune responses and mainly provides protection through tolerating self or foreign antigens (Doherty and O’Farrelly, 2000). When the tolerance is impaired, the activated immune cells could release pro-inflammatory cytokines and chemokines to induce liver injury and hepatic inflammation, which determines the severity of liver injury (Wang et al., 2015b). The regulatory immune cells, immune checkpoint molecules, and other immune factors may participate in the balance of immune activation and tolerance. Numerous studies have shown that their abnormalities may also contribute to the pathogenesis of DILI.

### Regulatory T Cells

Tregs maintain immune tolerance and homoeostasis of the immune system by inhibiting T cells activation and proliferation (Piccirillo and Shevach, 2001), blocking inflammatory cytokines release, and suppressing the immunoglobulins' production of B cells (Sakaguchi et al., 2010). CD4^+^CD25^+^ Tregs comprise approximately 5–10% of CD4^+^T cells in human peripheral blood (Roncador et al., 2005). A recent study has shown that the diminishment of Tregs induces the loss of immune privilege in the liver and rapidly initiates an inflammatory response that causes liver damage (Lu et al., 2013). Due to Tregs being capable of suppressing immune cell-mediated hepatocytes damage (Wei et al., 2008; Stross et al., 2012), their depletion could result in the aggravation of ALF. Studies have found that the number of Tregs and the expression of foxp3 in the liver were decreased significantly after TP treatment and that adoptive transfer of Tregs could improve TP-induced liver injury, while depletion of Tregs decreased the levels of IL-10 and aggravated liver injury with higher levels of ALT and AST (Czaja, 2015; Wang et al., 2016). Indeed, Tregs were also reported to alleviate APAP-induced liver injury through IL-10 and transforming growth factor-β1 (TGF-β) (Wang et al., 2015a). Tregs negatively regulated liver NKTs likely in an IL-10–dependent manner (Hua et al., 2011), and might also depend on disrupting the balance of T cells (Gao et al., 2019).

### Myeloid-Derived Suppressor Cells

Myeloid-derived suppressor cells (MDSCs) are heterogenous cells, which negatively regulate the immune system during infections, cancer, and other inflammatory conditions by direct cell–cell contact or secreting factors to suppress T cell responses (Gabrilovich and Nagaraj, 2009). MDSCs represent an intrinsic part of the myeloid–cell lineage comprised of the myeloid–cell progenitors and precursors of the myeloid cells. In pathological conditions, the activation of MDSCs could lead to the increased expression of immune suppressive factors such as inducible nitric oxide synthase, arginase, nitric oxide, and ROS. In addition, MDSCs not only have suppressive effects on adaptive immune responses but also show the regulation of innate immune responses via modulating the cytokine secretion from macrophages (Sinha et al., 2007). MDSCs infiltrate the liver and alleviate hepatotoxicity in experimental animal models of DILI (Liu et al., 2014). Furthermore, the depletion of hepatic MDSCs before halothane exposure could impair immune tolerance and aggravate liver injury (Chakraborty et al., 2015).

## Immune-Based Biomarkers and Drug-Induced Liver Injury

Traditionally, in clinical practice, the diagnosis of DILI is always started by accurately tracing the history of drug administration and liver biochemical abnormalities by serum levels of ALT, AST, ALP, γ-GT (European Association for the Study of the Liver, 2019), and TBIL through history taking from patients. And then, according to the DILI diagnostic tool Roussel Uclaf Causality Assessment Method (RUCAM), making a probabilistic decision by a score card. Several causality assessment methods and the recently updated RUCAM have been developed based on scores (Danan and Teschke, 2015). The causality score is limited in the great challenge to differentiate DILI from other liver injuries. Even though the EASL (European Association for the Study of the Liver, 2019) DILI guidelines proposed definitions for DILI, traditional biomarkers have poor specificity in distinguishing DILI from other liver injuries. Thus, currently the diagnosis of DILI is mainly based on clinical criteria and the elimination of other causes (Fontana et al., 2010). The levels of enzymes in the liver also have an insufficient correlation to histological patterns of DILI (Devarbhavi, 2012). Although these biomarkers are useful in severe DILI diagnosis for their functions to reflect hepatic lesions, they have many limitations making them not ideal as biomarkers. Some newly proposed biomarkers are promising for the early detection of DILI but are not yet available for routine use in clinical practices, and still need confirmation on their specificity, validity, and sensitivity, particularly in comparison to that of traditional ones.

### Human Leukocyte Antigen Polymorphisms

HLA, located on the human chromosome 6 short arm, is a gene complex composed of a series of tightly linked genes, encoding the MHC to regulate immunity. As aforementioned, a drug or its reactive metabolites such as haptens binding to proteins and then forming neoantigens, then presenting on specific HLA molecules, may initiate an inappropriate immune response that contributes to liver damage. The first successful genome-wide association study in DILI revealed flucloxacillin-induced liver injury with HLA-B*57:01 (Daly et al., 2009). Subsequent studies found that whites carrying HLA-B*35:02 were susceptible to minocycline-induced liver injury (Urban et al., 2017). Our research team (Li et al., 2019) had identified for the first time that HLA-B*35:01 was a specific risk gene for *P. multiflorum*–induced liver injury (Rodriguez-Pena et al., 2006; Megherbi et al., 2009). Growing evidences have revealed that individuals carrying certain class I and II *HLA* alleles are at increased risk of DILI (Kindmark et al., 2008; Daly et al., 2009; Singer et al., 2010; Lucena et al., 2011; Spraggs et al., 2011; Nicoletti et al., 2016; Parham et al., 2016; Nicoletti et al., 2017; Urban et al., 2017; Kaliyaperumal et al., 2018; Nicoletti et al., 2019). Despite the strong association with *HLA*, the positive predictive value of *HLA* allele polymorphisms in drug-induced adverse reactions is limited (Rao et al., 2020). These results suggest that there are other factors, other than the *HLA* allele, that contribute to the progression of IDILI. Despite a lot of research in this field, the precise activation of the immune system and how it effects liver injury remain to be deeply defined, such as extremely limited sample sizes, lack of prospective studies, randomized controlled trials, and excessive confounding factors.

### Antidrug Antibody

Idiosyncratic liver injury caused by drugs such as tienilic acid and halothane are associated with a variety of antibodies (Pohl et al., 1989; Bourdi et al., 1996; Robin et al., 1996), including antibodies against drug-modified proteins, anti-CYP antibodies, and other autoantibodies, as has been observed in DILI. The antibodies against amodiaquine (AQ) was detected in patients with liver injury induced by AQ, suggesting that AQ-induced idiosyncratic reactions represent an immune-mediated reaction against AQ-modified proteins (Christie et al., 1989). During therapy with nomifensine, specific IgE and IgG antibodies were identified (Wälti et al., 1983), and these findings may explain the immunological mechanisms of DILI and help in identifying patients at risk of serious adverse drug reactions.

### Exosomes

Recently, exosomes as extracellular vesicles from various cells, secreted as membranous structures, have been studied as an important tool for intercellular communication. Exosomes are usually characterized as small membrane vesicles (diameter: 40–150 nm; density: 1.10–1.18 g/ml) (Kowal et al., 2016). Interestingly, some studies have considered the exosomes as potential biomarkers for the early evaluation, monitoring, and detection of DILI (Bala et al., 2012; Cho et al., 2017; Royo et al., 2017). When primary human hepatocytes were exposed to APAP, the level of miR-122 was increased in exosomes (Holman et al., 2016). In animal experiments, transcriptome profiling analysis of circulating messenger RNAs showed that the circulating liver-specific mRNAs in exosomes have the potential to be biomarkers for the diagnosis of DILI (Wetmore et al., 2010). A study reported that the mRNA in exosomes may have a cytotoxic effect in traditional Chinese medicine (TCM), which suggested that exosomal miRNAs can be used to deeply understand the mechanisms of TCM-induced liver injury (Zheng et al., 2018). *In vivo* experiments further demonstrate that exosomes significantly increased the number of Treg and decreased pro-inflammatory cytokine IL-2, which plays a key role in immunosuppressive effect (Zhao et al., 2021). Interestingly, human-derived stem cell exosomes could significantly improve the liver function, by decreasing hepatic apoptosis and modulating IL-1β, IL-6, and TNF-α levels in the mouse model of acute liver injury (Chen et al., 2017). However, the limitations of exosomes including the poor understanding and unclear mechanisms should be clarified.

Recently, the research on diagnostic and predictive biomarkers of DILI has aroused the enthusiasm of researchers, and many biomarkers, such as microRNAs, HMGB1, glutamate dehydrogenase (GLDH), and keratin-18, have been discovered (Antoine et al., 2009). Howell et al. (2018) recently published a review highlighting miRNA-122 being greatly sensitive and specific in predicting and monitoring DILI. However, a multicenter study tested the performance of several biomarkers and found that GLDH was more valuable than miR-122 in diagnosing DILI (Church et al., 2019). In the liver, a cytoskeleton protein was increased early before ALT, leading to a real damage of hepatocytes, which may be a prognostic marker of liver injury (Church and Watkins, 2017; Church et al., 2019; Uetrecht, 2019). To date, several genome-wide association studies have been conducted in DILI, however, the biomarkers that can accurately predict DILI have not yet emerged (Nicoletti et al., 2017; Urban et al., 2017; Barnhill et al., 2018); however, these have not yet predicted biomarkers for identifying DILI accurately.

## Conclusion and Prospects

**FIGURE 2:**
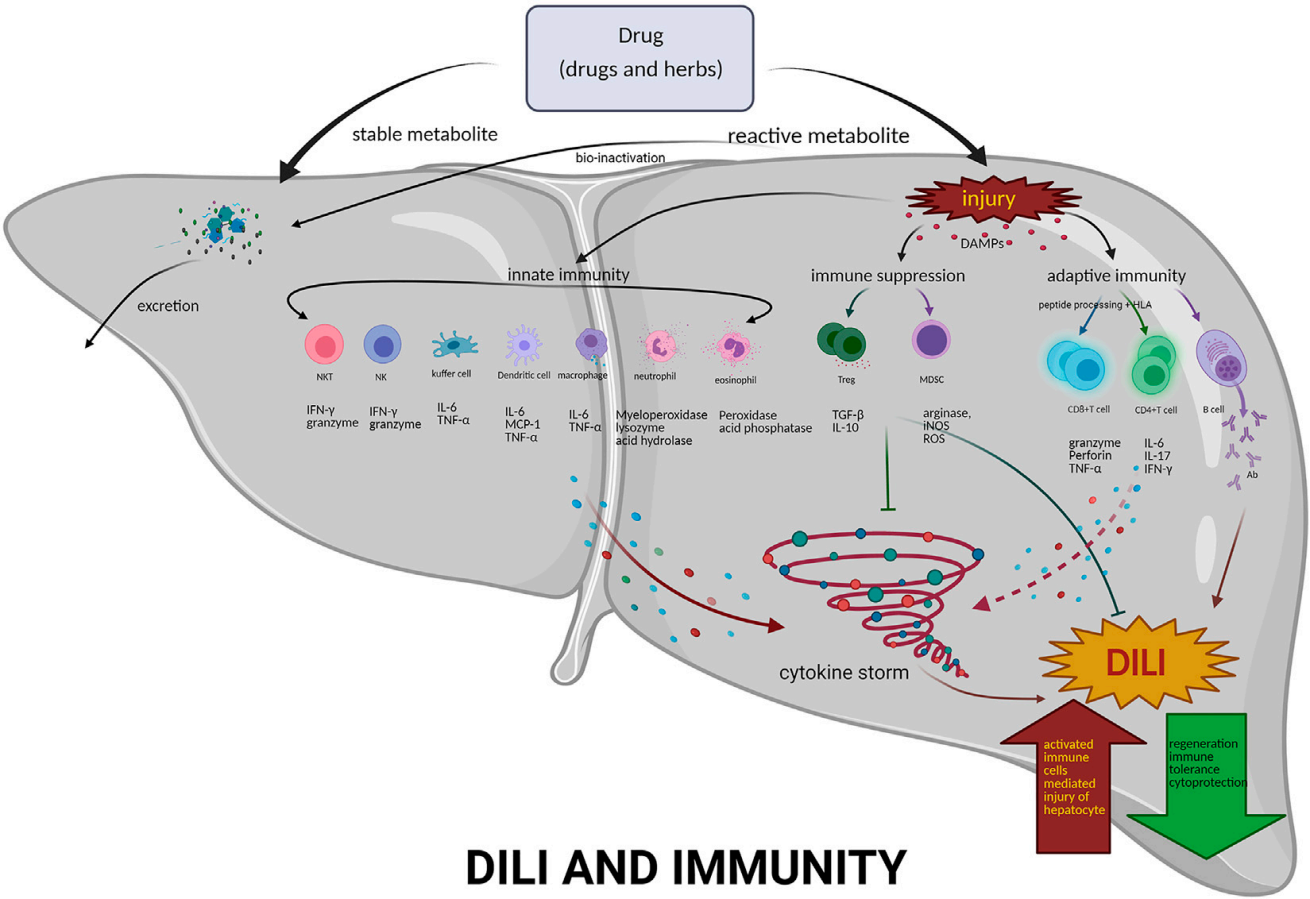
Mechanisms of immunity in DILI. DILI is a disorder by drugs-induced liver damage, including pharmacological therapies, traditional medicines, and HDS. If the number of reactive metabolites produced exceeds the detoxification capacity, activated innate immune system–related cells, e.g., NKT, NK, dendritic cells, Kupffer cells, neutrophils and eosinophils, and the released various cytokines, form the cytokine storm. Furthermore, inducing adaptive immunity, activating T and B cells, producing effector T cells and antibodies, then liver damage all occur. However, Tregs may exert an immunosuppressive effect on APCs, effector T cells, and mast cells by the following mechanisms: coinhibitory receptors binding to cognate molecules on dendritic cells, secretion of inhibitory cytokines, e.g., IL-10 and TGF-β, metabolic disruption by depriving IL-2 binding and increasing adenosine binding to effector T cells, and contact-dependent cytolysis by granzyme B secretion. Abbreviations: NKT, natural killer T cells; NK, natural killer cells; Treg, regulatory T cells; MDSC, myeloid-derived suppressor cells; DAMPs, danger-associated molecular patterns; IFN-γ, interferon gamma; IL-6, interleukin 6; TNF-α, tumor necrosis factor-α; IL-10 interleukin 10; TGF-β, transforming growth factor-β1; IL-17, interleukin 17; Ab, antibody; ROS, reactive oxygen species; iNOS, inducible nitric oxide synthase.

Clinicians mainly use exclusive diagnosis combed with causality assessment to improve DILI diagnostic, which is an important and arduous task for medical and health professionals. The pathological process of DILI is very complicated, and the specific mechanism has not been deeply elucidated mostly because of the lack of DILI animal models. The balance between immunity and tolerance is essential to liver function (Figure 2). Excessive inflammation may lead to liver injury, while insufficient immunity always allows for cancer or chronic infection. Dynamic interactions between the numerous subsets of immune cells in the liver are a key to DILI. Moreover, other factors involved in the immune response of DILI need to be clarified. A single biomarker is insufficient to accurately diagnose and predict the occurrence of DILI, thus more biomarkers need to be discovered and validated. Various new technologies of the fourth industrial revolution based on the combination of genomics, proteomics, and metabolomics will be developed and applied for the detection and diagnosis of DILI in the future.
